# “Treating myself first”: Healthcare-seeking experiences among migrant workers in Thailand’s fisheries sector

**DOI:** 10.1371/journal.pgph.0006765

**Published:** 2026-07-02

**Authors:** Niphattra Haritavorn

**Affiliations:** Faculty of Public Health, Thammasat University (Rangsit Campus), Pathum Thani, Thailand; University of Oxford, UNITED KINGDOM OF GREAT BRITAIN AND NORTHERN IRELAND

## Abstract

Migrant workers play a crucial role in Thailand’s fishery and seafood processing industry. However, their social and economic circumstances present significant barriers to healthcare access. This study explored the healthcare-seeking experiences of migrant workers employed in Thailand’s seafood processing sector. Data were collected through in-depth interviews with 30 migrant workers employed and five migrant health volunteers and analyzed using thematic analysis. In response to illness, participants navigated multiple healing systems through sequential healthcare-seeking practices described as taking care of myself first, turning to people I trust, using alternative treatments, and receiving biomedical services. Delayed healthcare seeking and reliance on self-medication were commonly shaped by financial insecurity, unstable employment, and concerns about income loss. Migrant health volunteers also played an important role in bridging linguistic, cultural, and social gaps between migrant communities and the formal healthcare system. These findings highlight how healthcare-seeking behaviors among migrant workers are shaped by broader social and structural conditions. Community-based interventions and migrant-inclusive health policies may help improve equitable access to healthcare services among migrant workers in Thailand.

## Introduction

Thailand was one of the major global producers and exporters of processed seafood by value in 2024, with exports totaling 7.08 billion USD [1]. Since seafood processing contributes vitally to the national economy, the industry relies heavily on migrant labor to address persistent domestic labor shortages [2]. According to the International Organization for Migration [3], there were 3,143,120 regular migrant workers in elementary occupations from Cambodia, the Lao People’s Democratic Republic, Myanmar, and Vietnam. The number is likely underestimated due to the presence of irregular workers who lack official documentation. In the fishery sector, the workforce is predominantly from Myanmar and Cambodia because of Thailand’s geographic proximity and shared land and maritime borders with these neighboring countries.

As a large proportion of migrants are employed in the fishery sector, many are excluded from formal healthcare services. This exclusion is particularly concerning given the significant occupational health risks associated with fisheries work. Many studies have shown that handing heavy loads and pulling unstable nets contribute to work-related musculoskeletal disorders affecting the lower back, shoulders, knees, and hands [4–6]. Similarly, aquaculture workers are exposed to a range of occupational health problems such as musculoskeletal disorders, respiratory symptoms and asthma, skin infections, dermatitis, and urticaria [7]. Moreover, the seafood processing stage introduces further biological hazards, leaving processing workers highly vulnerable to developing occupational allergic reactions affecting the lungs and skin [8].

Given their exclusion from formal healthcare services, these workers adopt independent strategies to manage their illness. Consequently, they navigate health practices ranging from self-medication to seeking care from alternative practitioners. Self-medication – defined as the selection and use of medicines to alleviate symptoms without formal clinical consultation – frequently serves as the foundation of these behaviors, offering a convenient and economical alternative to institutional care [9,10]. While self-medication offers immediate cost-mitigation, it also introduces significant risks, including incorrect diagnosis, hazardous dosing, and delays in seeking professional medical treatment [11,12].

While the risks of self-medication are recognized, healthcare decisions are often influenced by perceptions of illness causation, beliefs in the effectiveness of particular treatments, the cost of care, and previous experience with managing minor ailments [13]. Additionally, the decision-making processes of Mexican immigrants depend heavily on the nature and severity of illness [14]. Likewise, Wen et al. [15] note that Miao migrants frequently navigate a medically pluralistic system that integrate shamanistic ritual healing, herbalism, and folk biomedicine within a spiritually coherent framework rooted in traditional religion and cosmologies.

These health-seeking behaviors have been documented globally but they manifest under particularly precarious conditions within Thailand’s seafood sector. Notably, migrant fishery workers in Thailand experience severe work-related musculoskeletal disorders driven by heavy lifting nets, prolonged working hours, continuous exposure to harsh environmental conditions, and ergonomically hazardous postures [16]. As these demanding physical tasks are performed daily without adequate recovery time, temporary strains frequently progress into chronic and debilitating illnesses that threaten the workers’ livelihoods.

This high burden of workplace illness exists alongside barriers to formal medical care within Thailand. To address this issue, the Thai government established the Migrant Health Insurance Scheme to provide mandatory health examination, medical treatment, and communicable disease prevention [17,18]. However, despite these efforts, migrant workers continue to face barriers to healthcare utilization due to migration status, gender, language, discrimination, and economic constraints. [19]. These barriers reflect broader social and structural conditions that shape healthcare and influence healthcare-seeking decisions. As a result, many migrant workers rely on alternative healthcare-seeking strategies, including over-the-counter medicines and informal source of care, with healthcare-seeking practices further influenced by factors such as occupation, language fluency, injury severity, and chronic disease status [20,21].

Because qualitative explanations of health-seeking behaviors among migrant fishery workers in Thailand remain limited, this study aims to elucidate these behaviors within the framework of medical pluralism. By exploring workers’ lived experiences of healthcare-seeking behaviors, the study investigates how migrant workers navigate multiple forms of care and draw upon diverse sources of health knowledge. Understanding these experiences is important not only for identifying barriers to healthcare access but also for recognizing perspectives that remain underrepresented in global health research. The findings may inform more contextually responsive healthcare interventions and improve access to treatment and prevention programs by aligning them with the socio-cultural realities of migrant populations.

## Methods

### Ethics statement

Prior to the commencement of the study, ethical approval was obtained from the Human Research Ethics Committee of Thammasat University (Science), project code 66PU133. Informed consent was obtained from all participants before interviews were scheduled. Following a full explanation of the study, including interview duration and scope of questions, participants were asked to sign a written consent form, which was securely stored to ensure confidentiality. Participants were assured that all collected data would remain confidential, and pseudonyms were assigned to protect their identities throughout the research process. All individuals who agreed to participate completed the interviews, and no participants withdrew from the study.

### Study design

This study adopted a qualitative descriptive approach to understand the realities and experiences of healthcare seeking among migrant workers in Ranong province, Thailand. According to Doyle et al. [22], this methodology explores how the world appears to others and how individuals relate to their experiences and behaviors, thereby allowing researchers to describe the meaning of lived experiences. Using this framework, the study focuses on capturing the day-to-day realities of navigating healthcare services from the perspective of migrant fishery workers themselves.

In qualitative descriptive inquiry, in-depth interviews serve as the primary method of data collection [23]. In this study, all interviews were digitally recorded and supplemented with field notes to capture environmental and social context during analysis. An in-depth interview guide developed by the author was used. The guide was pilot tested with two migrant workers and refined accordingly. No repeat interviews were conducted. The researcher had no prior relationship with the participants before recruitment. Contact was facilitated through migrant health volunteers and community networks. With participants’ permission, interviews were conducted either at the participants’ homes or workplaces. Each interview lasted approximately 30–45 minutes. The interviews were conducted by the corresponding author, who is the sole researcher in this study (female) and a researcher with experience in qualitative research methods. The interview guide consisted of open-ended questions followed by targeted and clarifying questions regarding specific healthcare-seeking behaviors. Participants did not provide feedback on the findings.

Although all interviewed migrant workers were able to communicate in Thai and participate directly in the interviews, both migrant workers and migrant health volunteers frequently described barriers experienced by other migrant workers in the broader community, including undocumented migrants and those with limited Thai-language proficiency. These volunteers played an important role in bridging minor linguistic nuances, building rapport, and ensuring participants’ cultural comfort. Following data collection, the audio recordings were transcribed verbatim and analyzed to identify recurring themes and develop a comprehensive understanding of workers’ healthcare-seeking experiences. This study is reported in accordance with the Consolidated Criteria for Reporting Qualitative Research (COREQ) checklist (S1 Checklist).

### Setting

Ranong is a coastal province in southern Thailand on the Andaman coast and serves as a major gateway for Myanmar migrant workers as it is connected to Myanmar through overland border crossing. Due to the extensive length of the Thai-Myanmar border, Ranong had 34,423 registered Myanmar migrants as of March 2025. Myanmar migrants migrated to Ranong for several reasons that are better living conditions, the opportunity to acquire job skills in Thailand, political and economic unrest in Myanmar, and personal aspirations [24]. The local economy relies heavily on fishing and seafood processing, making Ranong one of the largest seafood markets in southern Thailand. Its marine and aquaculture sectors supply both domestic and international markets. Migrant workers play a crucial role in the province’s economy, particularly in the fishing and seafood processing sectors, which are major sources of employment for Myanmar migrants in Ranong.

### Sampling

This study included 30 Myanmar migrant workers and five Myanmar migrant health volunteers who were employed in Ranong Province, Thailand. Participants were recruited for the study from May 1, 2024 to August 31, 2024. No prior relationship existed between the researcher and participants before recruitment. Participants were recruited using purposive sampling with assistance from migrant health volunteers and community networks in Ranong Province. The inclusion criteria required participants to be 21 years of age or older, hold a government-issued document, possess Myanmar nationality, have lived in Thailand for more than three years, and have experience working in seafood processing. Methodologically, the study focused specifically on onshore seafood processing workers and excluded those employed directly aboard fishing vessels. The participants were engaged in pre- and primary seafood processing activities and worked in pier-based operations, seafood markets, processing facilities, and home-based workplaces. Their daily tasks involved loading fishing vessels as well as sorting, clearing, peeling, and drying seafood. Most participants worked as daily-wage or home-based workers under precarious employment arrangements, receiving compensation only when work was available. Notably, this study focused exclusively on documented migrant workers and did not include undocumented migrants at the time of the interviews. However, participants reported concerns related to documentation renewal, interactions with authorities, and employment-related restrictions that influenced healthcare-seeking behaviors. Participants also referred to the experiences of undocumented workers in their communities, which shaped their perceptions of healthcare access and potential risks associated with seeking care (Table 1).

**Table 1:** Socio-demographic characteristics of migrant worker participants (n = 30).

Characteristics	Category	Number
Age	21-30	7
	31-40	10
	41-50	8
	51-60	5
Gender	Male	21
	Female	9
Marital status	Single	3
	Married	23
	Other	4
Length of employment in sector	1-2 years	7
	3-4 years	13
	More than 4 years	10
Type of processing facility	Large-scale factory (export/canning)	5
	Small/Medium workshop (peeling shed)	25

### Analysis

**Fig 1:**
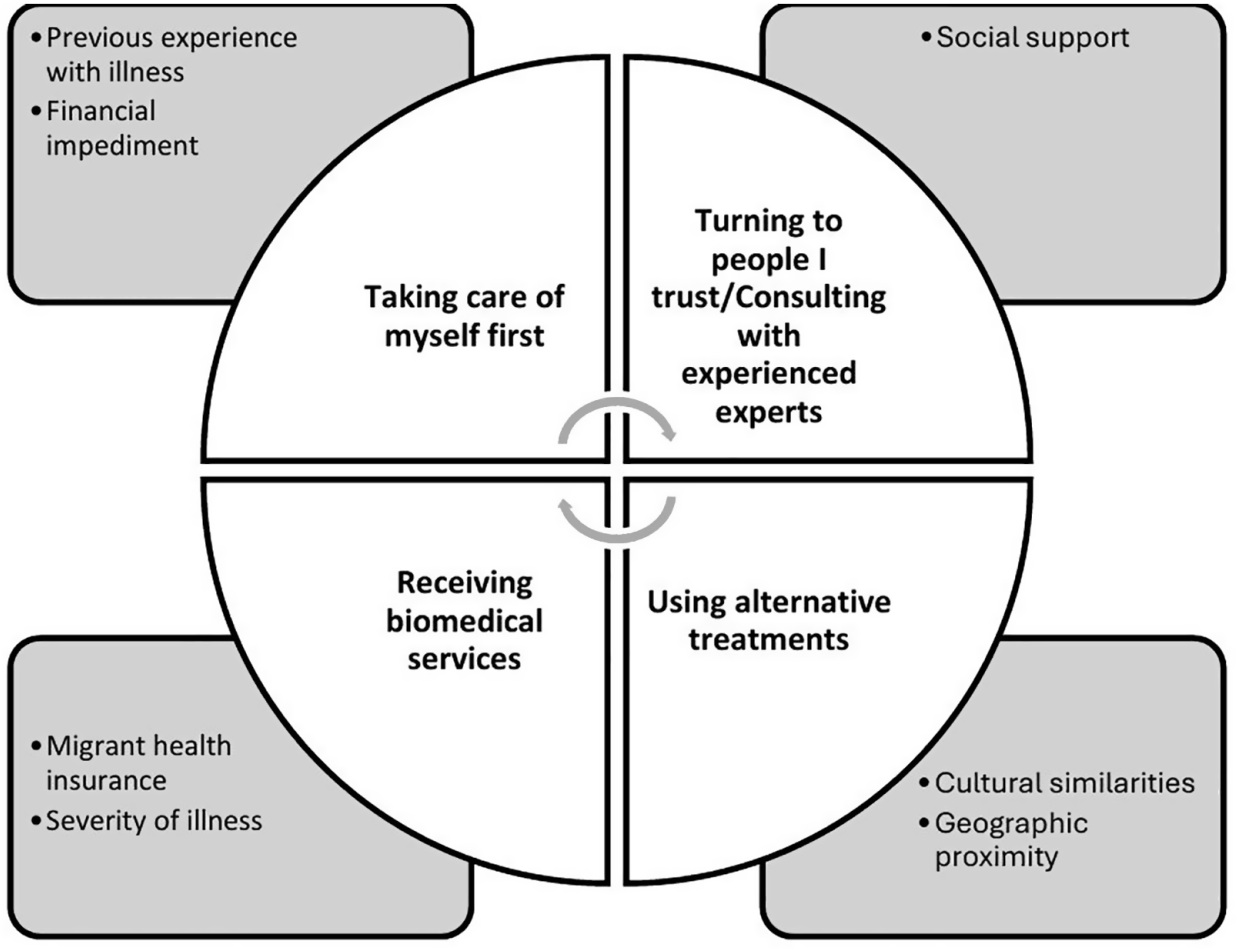
Process model of healthcare-seeking experiences among migrant workers.

The data were analyzed using thematic analysis following Clarke and Barun’s framework [25] to identify and interpret recurring themes across the interviews. All transcripts were coded by the corresponding author, and themes were derived inductively through iterative coding. Data were analyzed manually without qualitative software, and participants did not provide feedback on the findings. All interviews were transcribed verbatim in Thai within 72 hours of completion to maintain data integrity. Open coding was initially conducted to generate preliminary codes and identify emerging themes directly from the raw data. The codes were then grouped into categories and subcategories to refine and organize the thematic framework. Finally, these categories were organized into overarching themes that directly answered the research questions. Interview transcripts from migrant workers and migrant health volunteers were analysed together using the same thematic analysis framework. The perspectives of migrant health volunteers contributed to the development and refinement of themes and provided contextual insights that helped triangulate migrant workers’ accounts of healthcare-seeking experiences. The healthcare-seeking process identified in this study is summarized in Fig 1.

### Rigor

To ensure the rigor and trustworthiness of the findings, this study adopted the trustworthiness framework proposed by Lincoln and Guba [26], which includes credibility, transferability, dependability, and confirmability. Credibility was established through informal observations within the Myanmar community in Ranong and through collaboration with migrant health volunteers who helped ensure cultural sensitivity and accurate interpretation. Moreover, data saturation was achieved during the fieldwork by continuously comparing new interview data with earlier responses. The researcher found that no new codes, subcategories, or overarching themes emerged from the final interviews, signaling that data collection was complete. Transferability was supported by providing a thick description of the participants’ lived experiences within the socioeconomic and structural context of Thailand’s seafood processing sector. To ensure dependability, the researcher kept a complete audit trail including all audio files, interview transcripts, and notes on how coding decisions evolved. Confirmability was strengthened through the use of reflective field notes and the review of interview data immediately after each session to ensure that the findings reflected the participants’ perspectives rather than researcher assumptions. The researcher also reflected on how prior engagement with migrant communities could shape data interpretation throughout the study.

### Researcher positionality and reflexivity

As a qualitative researcher, I recognize that my background, identity, and experiences shape how the research is conducted and interpreted. I was trained in anthropology and am currently based in a Faculty of Public Health. These two disciplinary backgrounds inform the way I approach the data. Anthropology helps me attend to lived experience and context, while public health training directs attention toward health systems and policy relevance.

I have also worked extensively with migrant worker populations in previous qualitative studies. This long-term engagement helped me understand many of the structural and social conditions that shape migrant health experiences. At the same time, I remain conscious that this familiarity could influence how I interpret participants’ accounts, particularly when identifying patterns that seem consistent with earlier work. To address this, I tried to stay close to the data during coding and repeatedly checked my interpretations against the original transcripts.

Being an external researcher also meant that I had to be mindful of power relations during data collection. I therefore conducted interviews with the support of migrant health volunteers (MHVs) and in locations chosen by participants, which helped create a more relaxed and familiar environment. This arrangement also supported communication and reduced potential discomfort when discussing sensitive experiences.

Throughout the study, I kept field notes and used them alongside the transcripts during analysis to reflect on how my position and prior assumptions might be shaping interpretation. Themes were developed iteratively, and I returned to the raw data multiple times to ensure that the findings remained grounded in participants’ accounts, especially when describing how they navigated different healthcare systems.

### Single authorship

This study is single-authored. I was responsible for the study design, ethics applications, field coordination, data analysis, and manuscript preparation. Data collection was conducted with the support of migrant health volunteers, who facilitated communication and access to participants.

Single authorship allowed close engagement with the data throughout the research process but also meant that interpretations were developed from a single analytical perspective. To enhance rigor, I reviewed the interview data repeatedly, maintained reflexive notes during analysis, and considered how my background in anthropology and public health shaped the interpretation of the findings.

## Results

The following themes reflect the practical strategies employed by the participants to manage their illnesses. Their healthcare-seeking behaviors were not isolated actions but flexible responses that shifted according to their socioeconomic circumstances and structural constraints.

### Taking care of myself first

As workers in the seafood processing sectors, the participants frequently encountered workplace-related illnesses and physical ailments, including musculoskeletal disorders, colds, headache, cough, sore throat, diarrhea, and minor injuries. All the participants reported that upon experiencing initial symptoms, their immediate response was to address the illness independently through self-medication. Nyan Yun (51-year-old male) described this widespread reliance on self-medication:

“I had all the medicine at home to treat some common illnesses like muscle pain or a cold. When I was sick, I just took those medicines. It was just a common illness and I knew how to treat it. When my kids were sick, I just gave them the medicine. There was no need to go to the hospital.”

Musculoskeletal disorders – driven by physically demanding work such as loading fishing vessels and sorting, cleaning, peeling, and drying seafood – were among the most frequently mentioned conditions by the participants. They described pain in the low back, shoulders, and neck due to these repetitive and strenuous activities. To manage these symptoms, the participants commonly used muscle relaxants and painkillers. Nay Win (47-year-old male) mentioned his experience managing muscle pain:

“I have suffered from muscle pain for many years. It comes and goes quite often. When I have muscle pain, I would take ‘ya para’ (paracetamol) or use muscle relaxants. The symptoms disappear for a while, but then, the next month, the symptoms appear again. I think this is because I unload fishing vessels every day.”

Self-medication was commonly used for illnesses perceived as minor or less severe. However, this practice sometimes led to inappropriate use of unprescribed antibiotics. Because pharmaceutical drugs, especially antibiotics, are easily accessible in local drugstores without a prescription, participants routinely managed their symptoms independently. Min Min (31-year-old female) described this practice:

“When I get a cold, I go to pharmacies in the village to buy medicine. These include cold and sore throat medicines like antibiotics. The pharmacist prescribed me seven days sore throat medicine, but I only took it for three or four days. When the symptoms got better, I stopped taking the medicine and kept the rest for next time.”

Common accidents such as minor injuries resulting from knives or falling from the boats were described by Tin Shwe (27- year-old male) and Sai Saw (34-year-old female):

“The pier was wet and slippery. Although I wore boots, I slipped many times. Luckily, it was not serious, so I took some medicine and took a day off. I did not want to take many days off as I would not get paid. I just took a paracetamol and it would be fine.”

“I have to peel the shrimps. When a shrimp tail or shell poked my hand, I applied the medicine. It happened quite often because I work in this business. Having some injuries was not a big deal. If the wound was bleeding heavily and could not stop, I would go to see the doctor.”

Previous experiences with illness and past treatment outcome influenced participants’ reliance on self-medication. Based on their prior experiences, participants expressed confidence in managing similar conditions themselves. They believed that similar symptoms could be treated with drugs similar to those prescribed by medical doctors. Sia Sa Ma (35-year-old female) said:

“I did not want to go to the hospital. I went there once but the doctor just prescribed me some painkillers. It was just a simple drug that I could easily buy from Seven-Eleven (a convenient store). The drug costs around 20-30 baht (0.8 USD). If I went to the hospital, I would have to miss work, which meant I would lose my daily wage of 300 baht.”

Moreover, economic constraints and employment condition strongly influenced self-medication practices, as participants were concerned about losing daily wages of approximately 300–350 baht (10 USD) per day. Employment priorities often outweighed concerns about potential health risks. Zaw Oo (37-year-old male) said:

“I always cough because of an allergy. I went to see the doctor once. They prescribed me some medicine. The next time I had a cough, I brought this medicine to the pharmacy and bought the same kind. I did not have to go to see the doctor. It was a similar illness. The same drugs could also cure the illness.”

### Turning to people I trust/Consulting with experienced experts

Consultation with family, friends, and migrant health volunteers and experienced peers played an important role in health-seeking behavior as a form of social support. Experienced peers were crucial sources of health information and encouragement for seeking healthcare. U Min Aung (28-year-old male) said, “My friend noticed that I have got backache. He brought me some medicine. I did not know the name of drugs. He said he went to the clinic and the doctor prescribed him these drugs which, worked well.” Aung Yi (37-year-old male) often consulted his colleagues when he encountered muscle pain noting:

“When I get a cold, I would not go to the hospital. I would go to the clinic as my aunt recommended. This clinic injects drugs that lead to quick recovery. It took only one day to recover. It was much better than taking medicine. Many of my friends went to this clinic instead of going to the hospital.”

Migrant health volunteers provided health promotion and prevention within their communities, serving as a crucial link between the migrant populations and healthcare providers. Having received basic healthcare training, they became a focal point for community members seeking guidance. Su Su (43-year-old female), a migrant health volunteer, said:

“My job was like a bridge between healthcare workers and migrant workers in the community. The migrant workers here lived in their own communities and many of them could not speak Thai. They were afraid of going to the hospital. Therefore, I would suggest what they should do, and sometimes I would accompany them to the healthcare centers.

When participants became ill, they often consulted migrant health volunteers, who encouraged them to seek formal care and helped them navigate the healthcare system. Based on these consultations, participants decided whether to continue self-medication or receive medical treatments at a formal healthcare facility. Muang Maung (51-year-old female), a migrant health volunteer, said:

“Most of the migrant workers do not like to go to the hospital. Some of undocumented migrants in the community may be afraid they will get caught by the police or that they may lose money by taking a day off. Thus, I keep a first-aids kit or household medicines in my house. People in the community often stop by seeking healthcare. Just yesterday, a few children had diarrhea so they stopped by to ask for electrolyte powder.”

The volunteers actively helped participants navigate the healthcare services. Aye Hla (54-year-old male) who was diagnosed with colon cancer, said about this support:

“I have suffered from constipation since I was in Myanmar. At that time, I took a traditional herb as a laxative. When I migrated to Thailand, the symptom persisted. I went to a drugstore to buy medicine. One day I found blood in my stool. I talked to Yim (a health volunteer), and she told me I should go to the hospital immediately because this was a dangerous symptom.”

### Using alternative treatments

Sharing a border, Myanmar and Thailand have overlapping traditions in herbal medicine, with both nations relying heavily on local plants to treat illness. Participants familiar with both systems noted these similarities. Tee Woo (35-year-old male) recalled, “I saw that one of my colleagues took this leaf when he was fatigue. This leaf was similar to the ones in my hometown, so I asked where I could buy the leaves. This is a magical plant. It can cure many symptoms.” Fa Ti (43-year-old female) described the similarities between Thai and Burmese traditional medicine as follows:

“In Myanmar, when I got a cold, I usually took a medicine called, ‘Kyun Ywet Pon’ to relieve coughing. At first, I did not know where to buy it in Thailand. Then, a Thai helmsman gave me a Thai traditional herb to try. It was similar to the medicine in Myanmar so I thought they were similar herbs. Since then, I have preferred to buy this medicine in Thailand.”

The local market in downtown Ranong offers a wide array of medicinal herbs and traditional remedies from Myanmar. Beyond this market, participants also relied on visiting relatives from Myanmar to bring a supply of traditional medicine with them. Fa Yu (29-year-old female) noted, “When I wanted Myanmar drugs, I went to the market in downtown Ranong. There were plenty of Myanmar medicines there. If my parents visited me in Thailand, they usually brought some drugs with them.” Moreover, Ja Soo (51-year-old male) said:

“Many people think that it is difficult to find Myanmar herbs in Thailand, but this is not true. I found that there were similar herbs in the Thai markets, and even in the drugstores. The names are different, but they are the same plants. Some of my friends also brought some seeds from Myanmar and grew them in their gardens. They share the herbs with friends who are sick.”

Participants used herbal medicine because it reminded them of life in Myanmar. Some participants brought herb seeds from Myanmar and grew them in their rental houses in Thailand. Ma Thin Win (47-year-old female) said:

“I do not like taking western medicines. A nurse once told me that taking too many medicines can harm the liver. So, when I am sick, I use herbs from Myanmar. My great- grandparents were herbalists in Myanmar, and I inherited these traditional practices from them. I know how to use the herbs.”

Some participants used herbal medicine alongside biomedicine treatment because they believed that dual treatments increase effectiveness and speeded up recovery so they return to work more quickly. Tin Tin (45-year-old male) said:

“When I am sick, I take medicines from both Myanmar and Thailand. I buy the Myanmar medicine in the Thai market and the Thai medicine from a drugstore. If I get a cold, it is better to take herbs to relieve the cough whereas the tablets help me recover. Using both, it takes only two days to fully recover.”

### Receiving biomedical services

Notably, the participants were able to access health services at the provincial hospital through migrant health insurance, which allowed them to receive treatment free of charge. However, they emphasized that they only went to the hospital when their health problems could not be resolved in other ways. Daw Tin Tin (47-year-old female), who suffered from pneumonia mentioned:

“I had a cough for a while. I bought medicine from a drugstore but it did not clear it up My symptoms got worse so my husband told me I should go to the hospital. The doctor ordered a lung Xray. After looking at the film, he told me that I had pneumonia.”

The participants utilized hospital services as the severity of their illness increased. They delayed seeking hospital care when they perceived their conditions as mild and manageable through self-medication. Onn Yu Y (42-year-old female) mentioned:

“I had a headache, so I took paracetamol. The illness was resolved at first. But when it still persisted, I took more tablets. I used to take up to 10 tablets a day. The headache became disabling and I could not work, so I decided to go to the hospital. The doctor told me that a pain reliever like paracetamol could not cure my illness and that I needed to take migraine medicine.”

The majority of participants preferred private clinics because they perceived them as less complicated than public hospitals, although they had to pay out-of-pocket for treatment. Visiting a hospital usually took an entire day, while clinic visit typically lasted only one or two hours. Ko Ko (38-year-old male) said, “When my family and I were sick, we went to the clinic downtown. The clinic opens in the evening so I could go there after work. Because there were many Myanmar patients, some clinics even hired the Myanmar translator.” Ja (27-year-old male) mentioned:

“I usually go to a clinic. It is more convenient than going to the hospital. If I go to the hospital, I have to wake up at 6:00 AM to get a queue number. Then, I might not see the doctor until late morning or early afternoon, followed by waiting for payment and receiving prescribed drugs. It takes a whole day.”

## Discussion

Social realities profoundly shape the lived experiences of migrant workers seeking healthcare. In this study, fishery migrants’ health-seeking behaviors were shaped a dynamic interplay of social realities, values, beliefs, practices, and prior experiences. The findings show that the migrant workers sought care from a broad range of healthcare providers available within Thailand’s pluralistic medical system. Their healthcare decisions were influenced by economic precarity, social constraints, and perceived accessibility of services, often leading them to draw upon both formal and informal sources of care. By examining how migrant workers navigate multiple forms of care, this study contributes to a broader understanding of the diverse knowledge systems that inform healthcare decision-making among marginalized populations.

Whereas existing research has largely focused on how undocumented status and language barriers restrict healthcare access among migrant workers in Thailand [27], this study identified a more complex internal dynamic among documented workers. Despite possessing legal documentation, fishery migrant workers faced deeply entrenched financial impediments and broader social realities that delay access to appropriate biomedical treatments. These constraints limit engagement with formal health services, compelling them to rely on self-medication as a primary survival strategy. This practice is shaped by an intersection of educational backgrounds, the nature of illness, access to information, and personal beliefs and prior experience [28]. Furthermore, as documented in contexts like Greece, the structural nature of precarious, low-status, and low-wage jobs creates severe functional barriers to health services, [29,30]. Although language barriers are frequently reported as a major obstacle to healthcare access among migrant populations, this issue did not emerge as a dominant theme in our findings. Participants more commonly emphasized self-medication, work-related constraints, healthcare costs, and documentation concerns when describing their treatment-seeking decisions.

Moreover, previous experiences with illness lead participants to believe that they could manage their symptoms independently. Prior research has shown that self-medication is often associated with perceived cost-effectiveness, treatment efficacy, inefficiency within the healthcare system, and previous healthcare experiences reflecting the influence of social and contextual factors on healthcare-seeking behavior [31,32]. While Lei et al. [33] found that self-medication in China commonly involves managing acute or chronic conditions such as colds, coughs, cardiovascular problems, and gastrointestinal disorders, participants in this study primarily used self-medication to manage familiar and recurring symptoms, especially muscle pain or headaches. For these workers, previous experience served as a practical tool that helped them identifying recurring symptoms and navigate a complex healthcare system.

Moreover, this study found that health-seeking behavior is significantly shaped by social support networks, including health volunteers, family members, and peers. Thailand’s Migrant Health Volunteers (MHVs) framework has improved public health outcomes among migrant workers and played a vital role in responding to infectious diseases such as COVID-19 [34]. In this study, Migrant health volunteers function as indispensable cultural brokers, providing critical social support by bridging linguistic divides and helping migrants navigate the complexities of the Thai healthcare system.

Health beliefs and practices represent a society’s cultural worldview, manifested through activities designed to maintain health, protect against illness, and restore well-being [35]. This study highlights the ethnomedical similarities between Thailand and Myanmar, especially the widespread use of herbal remedies. Sharing a geographical border has fostered cross-border continuities in traditional medicine [36]. By contrast, the use of traditional and alternative treatments among migrants remains deeply rooted in ancestral Myanmar practices [37–39]. These behaviors are reinforced by the widespread availability of herbal remedies in local Thai markets.

Financial accessibility remains a global barrier to healthcare for migrant workers. For example, costs restrict access among migrants in Canada and China [40,41]. In contrast, migrant workers in Thailand are legally eligible for health insurance covering common illness at no individual cost. Despite this coverage, the everyday lived realities of these workers mean that the severity and acuity of an illness become key factors for accessing formal care. Seeking professional biomedical services becomes a final resort, initiated only after informal self-medication fails. In essence, this reliance on multiple healing systems manifests as pragmatic pluralism, enabling migrant workers to navigate illness dynamically without jeopardizing their daily livelihoods.

These compelling factors directly shape workers’ decisions regarding when and where to seek healthcare in Thailand. This finding aligns with Min et al. [42], who reported that healthcare utilization among Myanmar migrants in Chiang Rai is influenced by an intersection of age, financial capacity, working conditions, and health insurance coverage [42]. However, the findings of this study suggest that health-seeking behavior operates not only as an economic response to structural constraints, but also as part of a broader pluralistic medical system shaped by migrants’ lived experiences and cultural practices. The participants drew upon both biomedical and traditional paradigms in constructing their health strategies. Financial precarity nevertheless remained a key factor influencing treatment decisions and often delayed access to timely clinical care. These practices were further reinforced through the workers’ social networks, which served to sustain and legitimize parallel forms of healthcare within the migrant community.

Several limitations should be acknowledged. First, the findings may not be generalizable to the broader migrant population in Thailand because the sample was drawn exclusively from a specific group of registered Myanmar migrant workers, Furthermore, the sampling strategy was not intended to produce a statistically representative sample. Future research should therefore focus on unregistered migrant workers to better understand how a lack of legal status influences their health-seeking behaviors. Although interviews were conducted in Thai, variations in Thai language proficiency among participants may have influenced the depth and nuance of some responses.

## Conclusions

This study offers significant practical implications for migrant healthcare. Understanding the complexities of workers’ health-seeking behaviours requires recognition of the intersecting social, economic, and cultural forces that shape their lived experiences. Such recognition is necessary for designing effective public health interventions to be effective, especially in the planning, implementation, and evaluation of initiatives that are culturally grounded in the community’s existing perceptions and practices rather than externally imposed. In addition, improving healthcare access requires the dissemination of accessible health information through Migrant Health Volunteers (MHVs). Strengthening and expanding the MHV framework may therefore play a critical role in delivering health education, reducing the risks associated with self-medication, and promoting more equitable healthcare access for this vulnerable workforce.

## Supporting information

S1 Checklist COREQ Checklist. Consolidated criteria for reporting qualitative research checklist.
(PDF)
