# Supplementary material for: “Treating myself first”: Healthcare-seeking experiences among migrant workers in Thailand’s fisheries sector
Source: PLOS Glob Public Health. 2026 Jul 2;6(7):e0006765. doi: 10.1371/journal.pgph.0006765 (PMC13327187; doi:10.1371/journal.pgph.0006765)
Supplement: Attachment — Submitted filename: Response to reviewer.docx [file pgph.0006765.s003.docx]

**Dear Editor and Reviewers,**

I sincerely thank the Editor and Reviewers for their valuable comments and suggestions. I have carefully considered all comments and revised the manuscript accordingly. Detailed point-by-point responses to each comment, together with the corresponding revisions made in the manuscript, are provided below.

I appreciate the time and effort devoted to reviewing my manuscript and hope that the revisions satisfactorily address all concerns.

**Response to the Editor’s Comments**

| **Editor Comment** | **Response** | **Changes Made** |
| --- | --- | --- |
| The ethical approval number(s) listed in the manuscript and/or submission metadata does not match the approval number on the ethical approval document you provided. Please ensure that all approval numbers are correct. | The revised manuscript now contains the same ethics approval number as shown in the ethical approval document. | On page 11, as “…. obtained from the Human Research Ethics Committee of Thammasat University (Science), project code 66PU133.” |
| Please ensure that the CRediT author contributions listed for every co-author are completed accurately and in full.  At this stage, the following Authors require contributions: Niphattra Haritavorn. Please ensure that the full contributions of each author are acknowledged in the "Add/Edit/Remove Authors" section of our submission form. | Thank you for this note. As this manuscript has a single author, the CRediT author contributions for Niphattra Haritavorn have been completed in the submission system. | The CRediT author contributions for Niphattra Haritavorn have been completed in the submission system. |
| Please provide separate figure files in .tif or .eps format. | Separate figure files have been prepared and uploaded in the required format (.tif) in accordance with the journal's submission guidelines. | Separate figure files have been uploaded in the submission system. |
| Your manuscript is missing the following sections: Results. Please ensure these are present, and in the correct order, and that any references to subheadings in your main text are correct. An outline of the required sections can be consulted in our submission guidelines here: [LINK] | I reviewed the manuscript structure and ensured that all required sections are present and arranged according to the journal guidelines. The Results section has been clearly identified and placed in the appropriate location within the manuscript. | On page 11, I changed the title section from ‘findings’ to ‘results.’ I rearranged the structure: title page-abstract-methods-results-discussion- conclusion-acknowledgements-references-supporting information. |
| We have noticed that you have uploaded Supporting Information files, but you have not included a list of legends. Please add a full list of legends for your Supporting Information files after the references list. | Legends for all supporting information files have now been added after the References section of the manuscript. | Ethics approval letter and COREQ checklist have been uploaded as S1 and S2 supporting information. |
| We note that you have indicated that there are restrictions to data sharing for this study. For studies involving human research participant data or other sensitive data, we encourage authors to share de-identified or anonymized data. However, when data cannot be publicly shared for ethical reasons, we allow authors to make their data sets available upon request. For information on unacceptable data access restrictions, please see.  If there are ethical or legal restrictions on sharing a de-identified data set, please explain them in detail (e.g., data contain potentially identifying or sensitive patient information, data are owned by a third-party organization, etc.) and who has imposed them (e.g., a Research Ethics Committee or Institutional Review Board, etc.). Please also provide contact information for a data access committee, ethics committee, or other institutional body to which data requests may be sent.  If there are no restrictions, please upload the minimal anonymized data set necessary to replicate your study findings to a stable, public repository and provide us with the relevant URLs, DOIs, or accession numbers. Please see <http://www.bmj.com/content/340/bmj.c181.long> for guidelines on how to de-identify and prepare clinical data for publication. For a list of recommended repositories, please see <https://journals.plos.org/plosone/s/recommended-repositories>. You also have the option of uploading the data as Supporting Information files, but we would recommend depositing data directly to a data reposit  Please update your Data Availability statement in the submission form accordingly or if possible. | I have revised the Data Availability Statement to provide further details regarding the ethical restrictions on data sharing. Because the study is based on qualitative interviews with migrant workers and stakeholders in a small study setting, public release of the interview transcripts could pose a risk of participant re-identification despite de-identification procedures. Therefore, the data are available only upon reasonable request and subject to ethical review. Contact information for the Human Research Ethics Committee of Thammasat University (Science) has been provided in the revised statement. | Following is the revised data availability statement, “The dataset consists of qualitative interview transcripts and field notes from migrant workers and stakeholders in the fish and seafood processing sector. Because these personal narratives contain highly specific occupational and migration experiences within a small study setting, public release is restricted to protect participant anonymity. These restrictions were established under the protocol approved by the Human Research Ethics Committee of Thammasat University (Science). De-identified data may be requested on a case-by-case basis by contacting the Committee at ecsctu3@tu.ac.th. The coding framework, thematic summaries, and anonymized excerpts supporting the findings are included within the manuscript.” |
| In the second-to-last paragraph of the Introduction, you clearly outline why the constraints faced by migrant workers are multifaceted. My suggestion is to strengthen this paragraph by linking these constraints to their vulnerability, marginalization, and the multi-level challenges they face, which are often nuanced and, at times, systemic. You may wish to engage more extensively with the literature on these issues, either in the Introduction or the Discussion section. <https://pubmed.ncbi.nlm.nih.gov/40527526/> In the final paragraph of the Introduction, I suggest strengthening the rationale. Vulnerability and the under-recognition of lived experiences are widespread, sometimes emerging in both obvious and subtle forms. Reticence and avoidance associated with systemic challenges are also prevalent and warrant further discussion. This calls for greater exploration of lived experiences, inductive forms of insight (e.g., ethnomedical practices and herbal medicine use), and minority communities’ knowledge of illness, including ancestral practices and how they navigate healthcare. Strengthen the rationale by explaining why such explorations are critical to the global health knowledge system. You may then link and echo these themes in the Discussion section through your argument on medical pluralism and the need for accommodation of plural knowledge systems, without presenting them as monolithic or antagonistic. <https://gh.bmj.com/content/6/5/e005829> | I revised the Introduction to further emphasize migrant workers’ lived experiences, healthcare-seeking practices, and the social and structural factors shaping access to care. I also strengthened the study rationale regarding the importance of understanding diverse healthcare knowledge and practices among marginalized populations. In addition, I expanded the Discussion to further contextualize our findings within medical pluralism and incorporated the recommended BMJ Global Health reference to support the introduction. | On page 5, the revised two paragraphs are as following, “This high burden of workplace illness exists alongside barriers to formal medical care within Thailand. To address this issue, the Thai government established the Migrant Health Insurance Scheme to provide mandatory health examination, medical treatment, and communicable disease prevention [1, 2]. However, despite these efforts, migrant workers continue to face barriers to healthcare utilization due to migration status, gender, language, discrimination, and economic constraints. [3]. These barriers reflect broader social and structural conditions that shape healthcare and influence healthcare-seeking decisions. As a result, many migrant workers rely on alternative healthcare-seeking strategies, including over-the-counter medicines and informal source of care, with healthcare-seeking practices further influenced by factors such as occupation, language fluency, injury severity, and chronic disease status [4, 5].  Because qualitative explanations of health-seeking behaviors among migrant fishery workers in Thailand remain limited, this study aims to elucidate these behaviors within the framework of medical pluralism. By exploring workers’ lived experiences of s healthcare-seeking behaviors, the study investigates how migrant workers navigate multiple forms of care and draw upon diverse sources of health knowledge. Understanding these experiences is important not only for identifying barriers to healthcare access but also for recognizing perspectives that remain underrepresented in global health research. The findings may inform more contextually responsive healthcare interventions and improve access to treatment and prevention programs by aligning them with the socio-cultural realities of migrant populations.  On page 19, I add the following sentence, “By examining how migrant workers navigate multiple forms of care, this study contributes to a broader understanding of the diverse knowledge systems that inform healthcare decision-making among marginalized populations.” |

**Response to reviewers 1 and 2**

| **Reviewer Comment** | **Response** | **Changes Made** |
| --- | --- | --- |
| Major Comments and Critical Issues A. Inexplicable Legal Status Contradiction (Crucial Revision Needed) There is a profound logical contradiction regarding the legal documentation status of the participants that undermines the validity of the discussion: • On line 132, the text states: "Notably, this study focused exclusively on documented migrant workers and did not include undocumented migrants at the time of the interviews." • Yet, on lines 258–259, a Migrant Health Volunteer explicitly states: "Some of them may be afraid they will get caught by the police..." • On lines 420–422, the Data Availability statement restricts the transcripts because they contain "sensitive qualitative narratives regarding illegal, informal, or precarious healthcare-seeking behaviors..." • Finally, on lines 343–345, the discussion notes that this study identified a more complex dynamic among "documented workers. Regardless of legal status..." Fix: If the study sample explicitly excluded undocumented workers, explain why documented workers possessing valid government-issued papers are terrified of police apprehension. If some participants were irregular or undocumented, the text must be corrected to reflect this reality, as legal precarity completely alters health-seeking dynamics. | Thank you for this observation. I would like to clarify that all study participants were documented migrant workers at the time of interview. I reviewed the manuscript and revised sections that could be interpreted as implying undocumented status or illegal activities by participants, including the Data Availability Statement. The revised text clarifies that concerns regarding confidentiality arise from the sensitive nature of the qualitative interviews and the potential for indirect identification within a small occupational setting, rather than participant legal status. | On page8, I add the following sentence, “Notably, this study focused exclusively on documented migrant workers and did not include undocumented migrants at the time of the interviews. Nevertheless, participants reported concerns related to documentation renewal, interactions with authorities, and employment-related restrictions that influenced healthcare-seeking behaviour. Participants also referred to the experiences of undocumented workers in their communities, which shaped their perceptions of healthcare access and potential risks associated with seeking care.”  On page 15, I revised the sentence as a migrant health volunteer, said, “Most of the migrant workers do not like to go to the hospital. Some of undocumented migrants in the community may be afraid…”  I have revised the data availability as following, “The dataset consists of qualitative interview transcripts and field notes from migrant workers and stakeholders in the fish and seafood processing sector. Because these personal narratives contain highly specific occupational and migration…”  On page 19, I have revised the sentence as following, “Despite possessing legal documentation, fishery migrant workers faced deeply entrenched financial…” |
| B. Omission of Primary Socio-Demographic Data On line 134, the author inserts a placeholder: Table 1: Socio-demographics of the study participants (n=30). Please insert the table in the main body of the manuscript, not as a supporting information | Accordingly, Table 1 (Socio-demographic characteristics of study participants, n = 30) has been moved from the Supporting Information and is now presented in the main manuscript. | The table has been inserted in the main body of the manuscript on page 8-9. |
| Missing Methodological Specifics The methodology section reads too abstractly and needs concrete anchoring: - Interview Language and Mediation: On line 100, the author states that "all participants were able to communicate fluently in Thai." Yet, on lines 248–249, a volunteer observes that "many of them could not speak Thai. They were afraid of going to the hospital." Clarify if the 30 workers interviewed were an exception to the wider community, or if the MHVs actively translated portions of the interviews. - MHV Sample Processing: The sampling text notes that 5 Myanmar migrant health volunteers were recruited, but the analysis framework entirely glosses over how their responses were managed. Were they analyzed alongside the 30 workers, or treated as a separate key-informant dataset? | I have clarified in the Methods section that all migrant workers included in this study were able to communicate in Thai and were interviewed directly without translation. The observation by the migrant health volunteer referred to language barriers experienced by some migrant workers in the wider community rather than the study participants themselves. I have also revised the data analysis section to clarify that interviews with the five migrant health volunteers were analyzed alongside the migrant worker interviews using the same thematic analysis framework. Their perspectives contributed to theme development and provided contextual information that helped triangulate migrant workers’ accounts and strengthen the interpretation of the findings. | On page 6, I add the following sentences, “Although all interviewed migrant workers were able to communicate in Thai and participate directly in the interviews, participants and migrant health volunteers frequently described barriers experienced by other migrant workers in the broader community, including undocumented migrants and those with limited Thai-language proficiency.”  On page 9-10, I added the following sentences, “Interview transcripts from migrant workers and migrant health volunteers were analyzed together using the same thematic analysis framework. The perspectives of migrant health volunteers contributed to the development and refinement of themes and provided contextual insights that helped triangulate migrant workers’ accounts of healthcare-seeking experiences.” |
| Minor Comments and Textual Corrections A. Literature Review and Citations • Inconsistent Citation Formatting: The manuscript haphazardly jumps between numbered Vancouver-style citations (e.g., [1], [12, 13]) and author-date Harvard-style citations inline (e.g., Stevenson et al., 2003 on line 53; Wen et al., 2025 on line 56; T. Aung et al., 2009 on line 75; Min et al., 2026 on line 386). Fix: Uniformly convert all inline parenthetical references to match the numeric Vancouver style mandated by PLOS guidelines. | I have revised the manuscript to ensure that all citations consistently follow the Vancouver style required by PLOS. | Author-date citations (e.g., Stevenson et al., 2003; Wen et al., 2025; T. Aung et al., 2009; Min et al., 2026) have been replaced with the appropriate Vancouver-style numbered citations throughout the manuscript in accordance with PLOS guidelines. |
| Typographical and Grammatical Errors • Line 76: Missing a period after the parenthetical citation: ...during severe health crises (T. Aung et al., 2009) These practices... • Line 96: Typo in sentence flow: ...capture surroundings of social context... change to ...capture the environmental and social context... • Line 110: Typo/grammar: Myanmar migrant migrated to Ranong... change to Myanmar migrants migrated to Ranong... or Migrants from Myanmar relocated to Ranong... • Line 136: Typo in framework name: Clarke and Barun's framework should be corrected to Braun and Clarke's framework. • Line 273: Grammatical slip: ...when the was fatigue. should be ...when he suffered from fatigue. or ...when he was fatigued. Or add (sic) in the sentence • Line 280: Typo: ...preferred to but this medicine... should read ...preferred to buy this medicine... • Line 316: Typo: ...perceived their conditions as mile... should be ...perceived their conditions as mild... • Line 317: Fragmented typo: Onn Yu Y (42-year-old female) mentioned that Remove the trailing "that" and punctuate the introduction to the quote properly. • Line 319: Typo: ...a pian reliever like paracetamol... change to ...a pain reliever like paracetamol... • Line 328: Typo: ...some clinic even hired... change to ...some clinics even hired... • Line 403: Structural redundancy/typo: ...In addition, to improving healthcare access requires... Change to: In addition, improving healthcare access requires... | I have corrected all Typographical and Grammatical Errors. | Line 94, “notes to capture environmental and social context during analysis.”  Line 117, “Myanmar migrants migrated to Ranong”  According to, larke V, Braun V. Thematic analysis. The Journal of Positive Psychology. 2017;12(3):297-8. doi: 10.1080/17439760.2016.1262613.  Line 156, “following Clarke and Barun’s framework”  Line 302, “when he was fatigue”  Line 309, “I have preferred to buy this”  Line 345, “their conditions as mild and manageable”  Line 346, “Onn Yu Y (42-year-old female) mentioned:”  Line 350, “a pain reliever like paracetamol”  Line 357, “some clinics even hired”  Line 445, “In addition, improving” |
| This paper has strong potential and aligns perfectly with the scope of PLOS Global Public Health. The sequential narrative of healthcare-seeking under severe systemic constraint is compelling. However, the author must clean up the text, iron out the deep logical contradiction regarding documentation/fear of police, and seamlessly integrate the baseline participant table to ensure the study is ready for publication. | I have carefully revised the manuscript to improve clarity, consistency, and overall readability. I also reviewed and revised sections discussing documentation status and concerns about interactions with authorities to clarify that, although all participants were documented migrant workers at the time of interview, issues related to documentation renewal, administrative requirements, mobility restrictions, and perceived legal insecurity continued to influence healthcare-seeking decisions. In addition, I have strengthened the integration of participant characteristics by revising the presentation of Table 1 and incorporating relevant demographic information into the Results section to provide clearer context for the findings. | On page 8, I add the following sentences, “Notably, this study focused exclusively on documented migrant workers and did not include undocumented migrants at the time of the interviews. Nevertheless, participants reported concerns related to documentation renewal, interactions with authorities, and employment-related restrictions that influenced healthcare-seeking behaviour. Participants also referred to the experiences of undocumented workers in their communities, which shaped their perceptions of healthcare access and potential risks associated with seeking care.”  On page 15, I revised as a migrant health volunteer, said, “Most of the migrant workers do not like to go to the hospital. Some of undocumented migrants in the community may be afraid…”  I have revised the data availability.  On page 19, I have revised as following, “Despite possessing legal documentation, fishery migrant workers faced deeply entrenched financial…” |
| The authors have presented a clear and coherent introduction, supported by an adequate review of the existing literature. The study rationale is clearly established, and the research objectives are clearly stated. The qualitative methodology is well presented, with a clear description of the study design, participant recruitment, data collection through in-depth interviews, and analytical procedures. The results are presented in a well-organized manner. However, language barriers, which are commonly reported as a challenge among migrant workers when accessing healthcare services, were not identified or discussed in the findings. It would be helpful for the authors to clarify whether language barriers were not reported by participants or whether this issue may have been overlooked during data collection or analysis. The limitations section could be strengthened by acknowledging potential recall bias, as participants may not accurately remember past healthcare-seeking experiences. Additionally, the authors should discuss possible language translation and interpretation issues that may have influenced data collection, participant responses, or the interpretation of findings. | Although language barriers were considered during data collection and analysis, they did not emerge as a prominent theme in participants’ accounts. Instead, participants more frequently highlighted self-medication practices, work-related constraints, healthcare costs, and perceptions of illness severity. I have added a clarification in the Discussion to acknowledge that, while language barriers are commonly reported in the literature, they were not a salient finding in this study.  Moreover, I have revised the Limitations section to acknowledge the potential for recall bias, as participants may not have accurately remembered all past healthcare-seeking experiences. I also added a statement noting that, although interviews were conducted in Thai, variations in participants’ Thai language proficiency may have influenced the depth and nuance of some responses and, consequently, the interpretation of findings. | On page 19-20, I added the following sentence, “Although language barriers are frequently reported as a major obstacle to healthcare access among migrant populations, this issue did not emerge as a dominant theme in our findings. Participants more commonly emphasized self-medication, work-related constraints, healthcare costs, and documentation concerns when describing their treatment-seeking decisions”  On page 22, I added this sentences in second last paragraph, “Although interviews were conducted in Thai, variations in Thai language proficiency among participants may have influenced the depth and nuance of some responses.” According to PLOS’s structure, the limitation was included with discussion section. |
| While revising your submission, we strongly recommend that you use PLOS’s NAAS tool (<https://ngplosjournals.pagemajik.ai/artanalysis>) to test your figure files. NAAS can convert your figure files to the TIFF file type and meet basic requirements (such as print size, resolution), or provide you with a report on issues that do not meet our requirements and that NAAS cannot fix. | I have converted the figure file to the TIFF file type. | The figure file to the TIFF file type was uploaded in submission system. |
