# Supplementary material for: “Treating myself first”: Healthcare-seeking experiences among migrant workers in Thailand’s fisheries sector
Source: PLOS Glob Public Health. 2026 Jul 2;6(7):e0006765. doi: 10.1371/journal.pgph.0006765 (PMC13327187; doi:10.1371/journal.pgph.0006765)
Supplement: Attachment — Submitted filename: Response to editor.docx [file pgph.0006765.s004.docx]

**Dear Editors,**

I sincerely thank the Editor for your valuable comments and suggestions. I have carefully considered all comments and revised the manuscript accordingly. Detailed point-by-point responses to each comment, together with the corresponding revisions made in the manuscript, are provided below.

I appreciate the time and effort devoted to reviewing my manuscript and hope that the revisions satisfactorily address all concerns.

**Response to the Editor’s Comments**

| **Editor Comment** | **Response** | **Changes Made** |
| --- | --- | --- |
| If the reviewer comments include a recommendation to cite specific previously published works, please review and evaluate these publications to determine whether they are relevant and should be cited. There is no requirement to cite these works unless the editor has indicated otherwise.   Please review your reference list to ensure that it is complete and correct. If you have cited papers that have been retracted, please include the rationale for doing so in the manuscript text, or remove these references and replace them with relevant current references. Any changes to the reference list should be mentioned in the rebuttal letter that accompanies your revised manuscript. If you need to cite a retracted article, indicate the article’s retracted status in the References list and also include a citation and full reference for the retraction notice. | Thank you for these literature suggestions. After careful evaluation, I have addressed them as follows:   - Adhikari et al. (2021): This study has been incorporated into the revised manuscript, as its qualitative methodological approach and findings on healthcare-related decision-making are relevant to the present study. - **Adhikari et al. (2025) & Mishra et al. (2022):** I reviewed both publications regarding internalized racism and epistemic indebtedness. While they offer vital contributions to debates on global health power dynamics, they do not directly intersect with the core objectives or findings of this manuscript, which focuses on the healthcare-seeking experiences of migrant workers in Thailand's seafood processing sector. Therefore, these two references were not included nto the revised manuscript.   Finally, I carefully reviewed the reference list to ensure completeness, accuracy, and consistency with journal formatting requirements. I also verified that none of the references cited in the manuscript have been retracted. No retracted publications were identified. Any updates made to the reference list during revision have been incorporated into the revised manuscript and are noted in this rebuttal letter where relevant. | Line 417: Prior research has shown that self-medication is often associated with perceived cost-effectiveness, treatment efficacy, inefficiency within the healthcare system, and previous healthcare experiences reflecting the influence of social and contextual factors on healthcare-seeking behaviour [31, 32].  32. Adhikari B, Pokharel S, Raut S, Adhikari J, Thapa S, Paudel K, et al. Why do people purchase antibiotics over-the-counter? A qualitative study with patients, clinicians and dispensers in central, eastern and western Nepal. BMJ Glob Health. 2021;6: e005829. doi: 10.1136/bmjgh-2021-005829. |
| Please strengthen your revised additions with relevant references (see my original comments). Also, please add a sub-heading within methods section and explain your positionality, impacts it may have on interpretations, including rationales and reflections on why this is a single authored paper. | In response to the editor's suggestion, I further strengthened the newly added sections on researcher positionality, reflexivity, and single authorship by incorporating relevant methodological literature on qualitative research and reflexive practice. | *Researcher Positionality and Reflexivity*  As a qualitative researcher, I recognize that my background, identity, and experiences shape how the research is conducted and interpreted. I was trained in anthropology and am currently based in a Faculty of Public Health. These two disciplinary backgrounds inform the way I approach the data. Anthropology helps me attend to lived experience and context, while public health training directs attention toward health systems and policy relevance.  I have also worked extensively with migrant worker populations in previous qualitative studies. This long-term engagement helped me understand many of the structural and social conditions that shape migrant health experiences. At the same time, I remain conscious that this familiarity could influence how I interpret participants’ accounts, particularly when identifying patterns that seem consistent with earlier work. To address this, I tried to stay close to the data during coding and repeatedly checked my interpretations against the original transcripts.  Being an external researcher also meant that I had to be mindful of power relations during data collection. I therefore conducted interviews with the support of migrant health volunteers (MHVs) and in locations chosen by participants, which helped create a more relaxed and familiar environment. This arrangement also supported communication and reduced potential discomfort when discussing sensitive experiences.  Throughout the study, I kept field notes and used them alongside the transcripts during analysis to reflect on how my position and prior assumptions might be shaping interpretation. Themes were developed iteratively, and I returned to the raw data multiple times to ensure that the findings remained grounded in participants’ accounts, especially when describing how they navigated different healthcare systems.  *Single Authorship*  This study is single-authored. I was responsible for the study design, ethics applications, field coordination, data analysis, and manuscript preparation. Data collection was conducted with the support of migrant health volunteers, who facilitated communication and access to participants.  Single authorship allowed close engagement with the data throughout the research process but also meant that interpretations were developed from a single analytical perspective. To enhance rigor, I reviewed the interview data repeatedly, maintained reflexive notes during analysis, and considered how my background in anthropology and public health shaped the interpretation of the findings. |
