# Supplementary material for: “Treating myself first”: Healthcare-seeking experiences among migrant workers in Thailand’s fisheries sector
Source: PLOS Glob Public Health. 2026 Jul 2;6(7):e0006765. doi: 10.1371/journal.pgph.0006765 (PMC13327187; doi:10.1371/journal.pgph.0006765)
Supplement: Peer review history

# Peer review: Decision Letter 0

**DOI:** 10.1371/journal.pgph.0006765.r001

**Authors:** Bipin Adhikari

8 Jun 2026

PGPH-D-26-01961

“Treating Myself First”: Healthcare-Seeking Experiences among Migrant Workers in Thailand’s Fisheries Sector

PLOS Global Public Health

Dear Dr. Haritavorn,

Thank you for submitting your manuscript to PLOS Global Public Health. After careful consideration, we feel that it has merit but does not fully meet PLOS Global Public Health’s publication criteria as it currently stands. Therefore, we invite you to submit a revised version of the manuscript that addresses the points raised during the review process.

Please submit your revised manuscript by Jul 08 2026 11:59PM. If you will need more time than this to complete your revisions, please reply to this message or contact the journal office at globalpubhealth@plos.org. When you're ready to submit your revision, log on to https://www.editorialmanager.com/pgph/ and select the 'Submissions Needing Revision' folder to locate your manuscript file.

Please include the following items when submitting your revised manuscript:

- A letter that responds to each point raised by the editor and reviewer(s). You should upload this letter as a separate file labeled 'Response to Reviewers'.
- A marked-up copy of your manuscript that highlights changes made to the original version. You should upload this as a separate file labeled 'Revised Manuscript with Track Changes'.
- An unmarked version of your revised paper without tracked changes. You should upload this as a separate file labeled 'Manuscript'.

Guidelines for resubmitting your figure files are available below the reviewer comments at the end of this letter.

As the corresponding author, your ORCID iD is verified in the submission system and will appear in the published article. PLOS supports the use of ORCID, and we encourage all coauthors to register for an ORCID iD and use it as well. Please encourage your coauthors to verify their ORCID iD within the submission system before final acceptance, as unverified ORCID iDs will not appear in the published article. *Only*  the individual author can complete the verification step; PLOS staff *cannot*  verify ORCID iDs on behalf of authors.

We look forward to receiving your revised manuscript.

Kind regards,

Bipin Adhikari, MBBS, DTM&H, MCTM, MPH, DPhil

Academic Editor

PLOS Global Public Health

Journal Requirements:

1. The ethical approval number(s) listed in the manuscript and/or submission metadata does not match the approval number on the ethical approval document you provided. Please ensure that all approval numbers are correct.

2. Please ensure that the CRediT author contributions listed for every co-author are completed accurately and in full.

At this stage, the following Authors require contributions: Niphattra Haritavorn. Please ensure that the full contributions of each author are acknowledged in the "Add/Edit/Remove Authors" section of our submission form.

The list of CRediT author contributions may be found here: INSERT LINK

https://journals.plos.org/globalpublichealth/s/authorship#loc-author-contributions

3. Please provide separate figure files in .tif or .eps format.

For more information about figure files please see our guidelines:  LINK

https://journals.plos.org/globalpublichealth/s/figures

https://journals.plos.org/globalpublichealth/s/figures#loc-file-requirements

4. Your manuscript is missing the following sections: Results. Please ensure these are present, and in the correct order, and that any references to subheadings in your main text are correct. An outline of the required sections can be consulted in our submission guidelines here: [LINK]

https://journals.plos.org/globalpublichealth/s/submission-guidelines#loc-parts-of-a-submission

5. We have noticed that you have uploaded Supporting Information files, but you have not included a list of legends. Please add a full list of legends for your Supporting Information files after the references list.

6. We note that you have indicated that there are restrictions to data sharing for this study. For studies involving human research participant data or other sensitive data, we encourage authors to share de-identified or anonymized data. However, when data cannot be publicly shared for ethical reasons, we allow authors to make their data sets available upon request. For information on unacceptable data access restrictions, please see http://journals.plos.org/plosone/s/data-availability#loc-unacceptable-data-access-restrictions.

Before we proceed with your manuscript, please address the following prompts:

a) If there are ethical or legal restrictions on sharing a de-identified data set, please explain them in detail (e.g., data contain potentially identifying or sensitive patient information, data are owned by a third-party organization, etc.) and who has imposed them (e.g., a Research Ethics Committee or Institutional Review Board, etc.). Please also provide contact information for a data access committee, ethics committee, or other institutional body to which data requests may be sent.

b) If there are no restrictions, please upload the minimal anonymized data set necessary to replicate your study findings to a stable, public repository and provide us with the relevant URLs, DOIs, or accession numbers. Please see http://www.bmj.com/content/340/bmj.c181.long for guidelines on how to de-identify and prepare clinical data for publication. For a list of recommended repositories, please see https://journals.plos.org/plosone/s/recommended-repositories. You also have the option of uploading the data as Supporting Information files, but we would recommend depositing data directly to a data repository if possible.

Please update your Data Availability statement in the submission form accordingly.

If the reviewer comments include a recommendation to cite specific previously published works, please review and evaluate these publications to determine whether they are relevant and should be cited. There is no requirement to cite these works unless the editor has indicated otherwise.

Please review your reference list to ensure that it is complete and correct. If you have cited papers that have been retracted, please include the rationale for doing so in the manuscript text, or remove these references and replace them with relevant current references. Any changes to the reference list should be mentioned in the rebuttal letter that accompanies your revised manuscript. If you need to cite a retracted article, indicate the article’s retracted status in the References list and also include a citation and full reference for the retraction notice.

Additional Editor Comments (if provided):

Your work titled “Treating Myself First” is a bold contribution that asks stakeholders and the health system to re-examine overlooked aspects of treatment-seeking behaviour among migrant workers in Thailand. This is an interesting manuscript and offers important insights. In addition to the reviewers’ comments, I would like to offer some broader suggestions for improvement.

In the second-to-last paragraph of the Introduction, you clearly outline why the constraints faced by migrant workers are multifaceted. My suggestion is to strengthen this paragraph by linking these constraints to their vulnerability, marginalization, and the multi-level challenges they face, which are often nuanced and, at times, systemic. You may wish to engage more extensively with the literature on these issues, either in the Introduction or the Discussion section.

https://pubmed.ncbi.nlm.nih.gov/40527526/

In the final paragraph of the Introduction, I suggest strengthening the rationale. Vulnerability and the under-recognition of lived experiences are widespread, sometimes emerging in both obvious and subtle forms. Reticence and avoidance associated with systemic challenges are also prevalent and warrant further discussion. This calls for greater exploration of lived experiences, inductive forms of insight (e.g., ethnomedical practices and herbal medicine use), and minority communities’ knowledge of illness, including ancestral practices and how they navigate healthcare. Strengthen the rationale by explaining why such explorations are critical to the global health knowledge system.

https://jogh-ep.org/article/36869-epistemic-indebtedness-do-we-owe-to-epistemic-enterprises

You may then link and echo these themes in the Discussion section through your argument on medical pluralism and the need for accommodation of plural knowledge systems, without presenting them as monolithic or antagonistic.

https://gh.bmj.com/content/6/5/e005829

[Note: HTML markup is below. Please do not edit.]

Reviewers' comments:

Reviewer's Responses to Questions

**Comments to the Author**

1. Does this manuscript meet PLOS Global Public Health’s publication criteria? Is the manuscript technically sound, and do the data support the conclusions? The manuscript must describe methodologically and ethically rigorous research with conclusions that are appropriately drawn based on the data presented.

Reviewer #1: Yes

Reviewer #2: Yes

**********

2. Has the statistical analysis been performed appropriately and rigorously?

Reviewer #1: N/A

Reviewer #2: Yes

**********

3. Have the authors made all data underlying the findings in their manuscript fully available (please refer to the Data Availability Statement at the start of the manuscript PDF file)?

The PLOS Data policy requires authors to make all data underlying the findings described in their manuscript fully available without restriction, with rare exception. The data should be provided as part of the manuscript or its supporting information, or deposited to a public repository. For example, in addition to summary statistics, the data points behind means, medians and variance measures should be available. If there are restrictions on publicly sharing data—e.g. participant privacy or use of data from a third party—those must be specified.

Reviewer #1: Yes

Reviewer #2: Yes

**********

4. Is the manuscript presented in an intelligible fashion and written in standard English?

PLOS Global Public Health does not copyedit accepted manuscripts, so the language in submitted articles must be clear, correct, and unambiguous. Any typographical or grammatical errors should be corrected at revision, so please note any specific errors here.

Reviewer #1: Yes

Reviewer #2: Yes

**********

5. Review Comments to the Author

Please use the space provided to explain your answers to the questions above. You may also include additional comments for the author, including concerns about dual publication, research ethics, or publication ethics. (Please upload your review as an attachment if it exceeds 20,000 characters)

Reviewer #1: 1. Recommendation

Major Revision

2. General Overview

This manuscript utilizes a qualitative descriptive approach to explore the sequential, pluralistic healthcare-seeking pathways of 30 documented Myanmar migrant seafood-processing workers and 5 migrant health volunteers (MHVs) in Ranong Province, Thailand. The study addresses an essential public health issue—how socio-structural conditions, financial precarity, and cultural frameworks shape health behaviors in a historically marginalized and economically vital population.

The paper is well-grounded in literature, utilizes appropriate qualitative frameworks (Sandelowski, Lincoln & Guba, and Braun & Clarke), and features an intuitive process model (Figure 1). However, I am concerned about some specific issues in the manuscript that need to be addressed ( see below).

3. Major Comments and Critical Issues

A. Inexplicable Legal Status Contradiction (Crucial Revision Needed)

There is a profound logical contradiction regarding the legal documentation status of the participants that undermines the validity of the discussion:

• On line 132, the text states: "Notably, this study focused exclusively on documented migrant workers and did not include undocumented migrants at the time of the interviews."

• Yet, on lines 258–259, a Migrant Health Volunteer explicitly states: "Some of them may be afraid they will get caught by the police..."

• On lines 420–422, the Data Availability statement restricts the transcripts because they contain "sensitive qualitative narratives regarding illegal, informal, or precarious healthcare-seeking behaviors..."

• Finally, on lines 343–345, the discussion notes that this study identified a more complex dynamic among "documented workers. Regardless of legal status..."

Fix: If the study sample explicitly excluded undocumented workers, explain why documented workers possessing valid government-issued papers are terrified of police apprehension. If some participants were irregular or undocumented, the text must be corrected to reflect this reality, as legal precarity completely alters health-seeking dynamics.

B. Omission of Primary Socio-Demographic Data

On line 134, the author inserts a placeholder: Table 1: Socio-demographics of the study participants (n=30). Please insert the table in the main body of the manuscript, not as a supporting information

C. Missing Methodological Specifics

The methodology section reads too abstractly and needs concrete anchoring:

• Interview Language and Mediation: On line 100, the author states that "all participants were able to communicate fluently in Thai." Yet, on lines 248–249, a volunteer observes that "many of them could not speak Thai. They were afraid of going to the hospital." Clarify if the 30 workers interviewed were an exception to the wider community, or if the MHVs actively translated portions of the interviews.

• MHV Sample Processing: The sampling text notes that 5 Myanmar migrant health volunteers were recruited, but the analysis framework entirely glosses over how their responses were managed. Were they analyzed alongside the 30 workers, or treated as a separate key-informant dataset?

4. Minor Comments and Textual Corrections

A. Literature Review and Citations

• Inconsistent Citation Formatting: The manuscript haphazardly jumps between numbered Vancouver-style citations (e.g., [1], [12, 13]) and author-date Harvard-style citations inline (e.g., Stevenson et al., 2003 on line 53; Wen et al., 2025 on line 56; T. Aung et al., 2009 on line 75; Min et al., 2026 on line 386). Fix: Uniformly convert all inline parenthetical references to match the numeric Vancouver style mandated by PLOS guidelines.

B. Typographical and Grammatical Errors

• Line 76: Missing a period after the parenthetical citation: ...during severe health crises (T. Aung et al., 2009) These practices...

• Line 96: Typo in sentence flow: ...capture surroundings of social context... change to ...capture the environmental and social context...

• Line 110: Typo/grammar: Myanmar migrant migrated to Ranong... change to Myanmar migrants migrated to Ranong... or Migrants from Myanmar relocated to Ranong...

• Line 136: Typo in framework name: Clarke and Barun's framework should be corrected to Braun and Clarke's framework.

• Line 273: Grammatical slip: ...when the was fatigue. should be ...when he suffered from fatigue. or ...when he was fatigued. Or add (sic) in the sentence

• Line 280: Typo: ...preferred to but this medicine... should read ...preferred to buy this medicine...

• Line 316: Typo: ...perceived their conditions as mile... should be ...perceived their conditions as mild...

• Line 317: Fragmented typo: Onn Yu Y (42-year-old female) mentioned that Remove the trailing "that" and punctuate the introduction to the quote properly.

• Line 319: Typo: ...a pian reliever like paracetamol... change to ...a pain reliever like paracetamol...

• Line 328: Typo: ...some clinic even hired... change to ...some clinics even hired...

• Line 403: Structural redundancy/typo: ...In addition, to improving healthcare access requires... Change to: In addition, improving healthcare access requires...

5. Concluding Assessment

This paper has strong potential and aligns perfectly with the scope of PLOS Global Public Health. The sequential narrative of healthcare-seeking under severe systemic constraint is compelling. However, the author must clean up the text, iron out the deep logical contradiction regarding documentation/fear of police, and seamlessly integrate the baseline participant table to ensure the study is ready for publication.

Reviewer #2: The authors have presented a clear and coherent introduction, supported by an adequate review of the existing literature. The study rationale is clearly established, and the research objectives are clearly stated.

The qualitative methodology is well presented, with a clear description of the study design, participant recruitment, data collection through in-depth interviews, and analytical procedures.

The results are presented in a well-organized manner. However, language barriers, which are commonly reported as a challenge among migrant workers when accessing healthcare services, were not identified or discussed in the findings. It would be helpful for the authors to clarify whether language barriers were not reported by participants or whether this issue may have been overlooked during data collection or analysis.

The limitations section could be strengthened by acknowledging potential recall bias, as participants may not accurately remember past healthcare-seeking experiences. Additionally, the authors should discuss possible language translation and interpretation issues that may have influenced data collection, participant responses, or the interpretation of findings.

**********

6. PLOS authors have the option to publish the peer review history of their article (what does this mean?). If published, this will include your full peer review and any attached files.

**Do you want your identity to be public for this peer review?**  If you choose “no”, your identity will remain anonymous but your review may still be made public.

For information about this choice, including consent withdrawal, please see our Privacy Policy.

Reviewer #1: **Yes:** Shailendra Prasad

Reviewer #2: No

**********

[NOTE: If reviewer comments were submitted as an attachment file, they will be attached to this email and accessible via the submission site. Please log into your account, locate the manuscript record, and check for the action link "View Attachments". If this link does not appear, there are no attachment files.]

Figure Resubmissions:

While revising your submission, we strongly recommend that you use PLOS’s NAAS tool (https://ngplosjournals.pagemajik.ai/artanalysis) to test your figure files. NAAS can convert your figure files to the TIFF file type and meet basic requirements (such as print size, resolution), or provide you with a report on issues that do not meet our requirements and that NAAS cannot fix.

After uploading your figures to PLOS’s NAAS tool - https://ngplosjournals.pagemajik.ai/artanalysis, NAAS will process the files provided and display the results in the "Uploaded Files" section of the page as the processing is complete. If the uploaded figures meet our requirements (or NAAS is able to fix the files to meet our requirements), the figure will be marked as "fixed" above. If NAAS is unable to fix the files, a red "failed" label will appear above. When NAAS has confirmed that the figure files meet our requirements, please download the file via the download option, and include these NAAS processed figure files when submitting your revised manuscript.

# Author comment: Author response to Decision Letter 1

**DOI:** 10.1371/journal.pgph.0006765.r002

12 Jun 2026

Attachment Submitted filename: Response to reviewer.docx

# Editor report: Decision Letter 1

**DOI:** 10.1371/journal.pgph.0006765.r003

**Authors:** Bipin Adhikari

18 Jun 2026

PGPH-D-26-01961R1

“Treating Myself First”: Healthcare-Seeking Experiences among Migrant Workers in Thailand’s Fisheries Sector

PLOS Global Public Health

Dear Dr. Haritavorn,

Thank you for submitting your manuscript to PLOS Global Public Health. After careful consideration, we feel that it has merit but does not fully meet PLOS Global Public Health’s publication criteria as it currently stands. Therefore, we invite you to submit a revised version of the manuscript that addresses the points raised during the review process.

Please submit your revised manuscript by Jul 18 2026 11:59PM. If you will need more time than this to complete your revisions, please reply to this message or contact the journal office at globalpubhealth@plos.org. When you're ready to submit your revision, log on to https://www.editorialmanager.com/pgph/ and select the 'Submissions Needing Revision' folder to locate your manuscript file.

Please include the following items when submitting your revised manuscript:

- A letter that responds to each point raised by the editor and reviewer(s). You should upload this letter as a separate file labeled 'Response to Reviewers'.
- A marked-up copy of your manuscript that highlights changes made to the original version. You should upload this as a separate file labeled 'Revised Manuscript with Track Changes'.
- An unmarked version of your revised paper without tracked changes. You should upload this as a separate file labeled 'Manuscript'.

Guidelines for resubmitting your figure files are available below the reviewer comments at the end of this letter.

As the corresponding author, your ORCID iD is verified in the submission system and will appear in the published article. PLOS supports the use of ORCID, and we encourage all coauthors to register for an ORCID iD and use it as well. Please encourage your coauthors to verify their ORCID iD within the submission system before final acceptance, as unverified ORCID iDs will not appear in the published article. *Only*  the individual author can complete the verification step; PLOS staff *cannot*  verify ORCID iDs on behalf of authors.

We look forward to receiving your revised manuscript.

Kind regards,

Bipin Adhikari, MBBS, DTM&H, MCTM, MPH, DPhil

Academic Editor

PLOS Global Public Health

Journal Requirements:

If the reviewer comments include a recommendation to cite specific previously published works, please review and evaluate these publications to determine whether they are relevant and should be cited. There is no requirement to cite these works unless the editor has indicated otherwise.

Please review your reference list to ensure that it is complete and correct. If you have cited papers that have been retracted, please include the rationale for doing so in the manuscript text, or remove these references and replace them with relevant current references. Any changes to the reference list should be mentioned in the rebuttal letter that accompanies your revised manuscript. If you need to cite a retracted article, indicate the article’s retracted status in the References list and also include a citation and full reference for the retraction notice.

Additional Editor Comments (if provided):

The revised manuscript shows significant improvement, well done.

Please strengthen your revised additions with relevant references (see my original comments).

Also, please add a sub-heading within methods section and explain your positionality, impacts it may have on interpretations, including rationales and reflections on why this is a single authored paper.

[Note: HTML markup is below. Please do not edit.]

Reviewers' comments:

[NOTE: If reviewer comments were submitted as an attachment file, they will be attached to this email and accessible via the submission site. Please log into your account, locate the manuscript record, and check for the action link "View Attachments". If this link does not appear, there are no attachment files.]

Figure Resubmissions:

While revising your submission, we strongly recommend that you use PLOS’s NAAS tool (https://ngplosjournals.pagemajik.ai/artanalysis) to test your figure files. NAAS can convert your figure files to the TIFF file type and meet basic requirements (such as print size, resolution), or provide you with a report on issues that do not meet our requirements and that NAAS cannot fix.

After uploading your figures to PLOS’s NAAS tool - https://ngplosjournals.pagemajik.ai/artanalysis, NAAS will process the files provided and display the results in the "Uploaded Files" section of the page as the processing is complete. If the uploaded figures meet our requirements (or NAAS is able to fix the files to meet our requirements), the figure will be marked as "fixed" above. If NAAS is unable to fix the files, a red "failed" label will appear above. When NAAS has confirmed that the figure files meet our requirements, please download the file via the download option, and include these NAAS processed figure files when submitting your revised manuscript.

# Author comment: Author response to Decision Letter 2

**DOI:** 10.1371/journal.pgph.0006765.r004

21 Jun 2026

Attachment Submitted filename: Response to editor.docx

# Editor report: Decision Letter 2

**DOI:** 10.1371/journal.pgph.0006765.r005

**Authors:** Bipin Adhikari

22 Jun 2026

“Treating Myself First”: Healthcare-Seeking Experiences among Migrant Workers in Thailand’s Fisheries Sector

PGPH-D-26-01961R2

Dear Associate Professor Haritavorn,

We are pleased to inform you that your manuscript '“Treating Myself First”: Healthcare-Seeking Experiences among Migrant Workers in Thailand’s Fisheries Sector' has been provisionally accepted for publication in PLOS Global Public Health.

Before your manuscript can be formally accepted you will need to complete some formatting changes, which you will receive in a follow up email. A member of our team will be in touch with a set of requests.

Please note that your manuscript will not be scheduled for publication until you have made the required changes, so a swift response is appreciated.

IMPORTANT: The editorial review process is now complete. PLOS will only permit corrections to spelling, formatting or significant scientific errors from this point onwards. Requests for major changes, or any which affect the scientific understanding of your work, will cause delays to the publication date of your manuscript.

If your institution or institutions have a press office, please notify them about your upcoming paper to help maximize its impact. If they'll be preparing press materials, please inform our press team as soon as possible -- no later than 48 hours after receiving the formal acceptance. Your manuscript will remain under strict press embargo until 2 pm Eastern Time on the date of publication. For more information, please contact globalpubhealth@plos.org.

Thank you again for supporting Open Access publishing; we are looking forward to publishing your work in PLOS Global Public Health.

Best regards,

Bipin Adhikari, MBBS, DTM&H, MCTM, MPH, DPhil

Academic Editor

PLOS Global Public Health

***********************************************************

Reviewer Comments (if any, and for reference):
